# The genetic structure of the Belgian population

**DOI:** 10.1186/s40246-018-0136-8

**Published:** 2018-02-02

**Authors:** Jimmy Van den Eynden, Tine Descamps, Els Delporte, Nancy H. C. Roosens, Sigrid C. J. De Keersmaecker, Vanessa De Wit, Joris Robert Vermeesch, Els Goetghebeur, Jean Tafforeau, Stefaan Demarest, Marc Van den Bulcke, Herman Van Oyen

**Affiliations:** 1 Scientific Institute of Public Health, Brussels, Belgium; 2 Department of Medical Biochemistry and Cell Biology, Institute of Biomedicine, The Sahlgrenska Academy, University of Gothenburg, Gothenburg, Sweden; 3 Laboratory of Cytogenetics and Genome Research, Department of Human Genetics, KU Leuven, Leuven, Belgium; 4 Department of Applied Mathematics, Computer Science and Statistics, Ghent University, Ghent, Belgium; 5 Department of Public Health, Ghent University, Ghent, Belgium

**Keywords:** Genetic variability, Population genomics, Public health genomics

## Abstract

**Background:**

National and international efforts like the 1000 Genomes Project are leading to increasing insights in the genetic structure of populations worldwide. Variation between different populations necessitates access to population-based genetic reference datasets. These data, which are important not only in clinical settings but also to potentiate future transitions towards a more personalized public health approach, are currently not available for the Belgian population.

**Results:**

To obtain a representative genetic dataset of the Belgian population, participants in the 2013 National Health Interview Survey (NHIS) were invited to donate saliva samples for DNA analysis. DNA was isolated and single nucleotide polymorphisms (SNPs) were determined using a genome-wide SNP array of around 300,000 sites, resulting in a high-quality dataset of 189 samples that was used for further analysis. A principal component analysis demonstrated the typical European genetic constitution of the Belgian population, as compared to other continents. Within Europe, the Belgian population could be clearly distinguished from other European populations. Furthermore, obvious signs from recent migration were found, mainly from Southern Europe and Africa, corresponding with migration trends from the past decades. Within Belgium, a small north-west to south-east gradient in genetic variability was noted, with differences between Flanders and Wallonia.

**Conclusions:**

This is the first study on the genetic structure of the Belgian population and its regional variation. The Belgian genetic structure mirrors its geographic location in Europe with regional differences and clear signs of recent migration.

**Electronic supplementary material:**

The online version of this article (10.1186/s40246-018-0136-8) contains supplementary material, which is available to authorized users.

## Background

After the completion of the Human Genome Project in 2003, international efforts were initiated to map human genetic variation between populations. This variation has been described for 26 populations worldwide via the 1000 Genomes Project [1, 2]. While this is a valuable resource for studying global genetic variation, both the number of samples per population and the total number of populations studied are relatively low. To gain sufficient statistical power and avoid false positives/negatives due to unmatched control populations in genotype-phenotype association studies, population-based genetic reference data are required from more specific and extended populations [3]. To address this, population-based whole genome sequencing initiatives have been performed at the national level throughout Europe [4–7]. From these genetic population studies, it has become clear that there is a strong correlation between the geographical location and the genetic structure of different populations [8, 9], also at the more regional level (e.g., along a north-south axis in the Netherlands) [6, 7, 9, 10]. Currently, this genetic information is not available for the Belgian population.

Therefore, a population-based cross-sectional study, called BelPHG-21 (Belgian Public Health Genomics in the twenty-first century), was organized that aims at describing the genetic variability in the Belgian population and relating this variability to several indicators of health and disease to anticipate a future transition towards precision public health. To accomplish this, the BelPHG-21 study was organized in the context of the 2013 Belgian National Health Interview Survey (NHIS). The NHIS is a population-based survey that has been periodically organized since 1997. It has the purpose to assess the health status and its distribution in the population and to describe the association of this health status with its main determinants [11].

Here, we report on the BelPHG-21 results related to the genetic structure of the Belgian population. By incorporating NHIS information about the study subject’s residence and parental country of birth, we demonstrate small but clear genetic differences between different regions and illustrate how the population’s genetic structure is shaped by recent migration waves.

## Results

### Factors determining consent for former NHIS participants to participate in the BelPHG-21 population genetic study

**Fig. 1:**
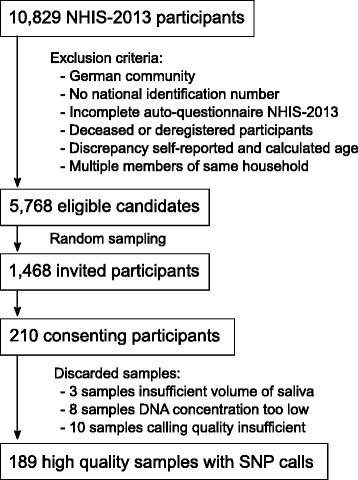
BelPHG-21 study flow chart, Belgium 2016

To study the genetic structure and variability in the Belgian population, a subset of participants from the most recent NHIS, conducted on 10,829 inhabitants in 2013 [12], was invited to donate saliva samples for DNA analysis (Fig. 1). From the invited subsample of 1468 individuals, 210 (14%) consented and submitted saliva samples for DNA analysis. Using the NHIS information related to demographics, education, employment, income, health behavior, lifestyle characteristics, physical and social environment, and wellbeing characteristics, we applied a predictive weighted binomial regression analysis to identify the factors that determined consent to participate in this study. This resulted in a model containing four variables (smoking behavior, education, age, and region of residence) (Table 1). The odds for consent were higher in non-smokers (and higher in former smokers than never smokers), higher age, and higher educational attainment. Furthermore, the likelihood for consent was also higher in the Flemish Region compared to the other regions.

**Table 1:** Variables predicting consent, BelPHG-21 study, Belgium 2016

Variable	Parameter	OR	95% CI OR
Smoking habits (ref “Smoker”)	Former smoker	2.21	[1.38, 3.62]
	Never smoker	1.38	[0.92, 2.15]
Age	Age (years)	1.02	[1.01, 1.02]
Residence (ref “Flanders”)	Brussels’ region	0.45	[0.24, 0.77]
	Walloon region	0.58	[0.41, 0.80]
Education (ref “No diploma”)	Lower secondary	2.29	[1.38, 7.02]
	Higher secondary	4.13	[2.12, 9.50]
	Higher	6.66	[3.40, 15.44]
	Not applicable	5.31	[2.25, 14.06]

A weighted binomial regression analysis to predict consent contained four variables (smoking habits, age, residence, and education). For each parameter, the odds ratio (OR) and 95% CI interval are given

### DNA sampling from the Belgian population

DNA was extracted from saliva samples obtained from 210 consenting participants. Three of these samples contained insufficient saliva, while the DNA concentration of eight other samples was considered too low for downstream analysis (Fig. 1). From the remaining 199 samples, single nucleotide polymorphisms (SNPs) at ± 300,000 sites were measured using the whole genome scanning Illumina HumanCytoSNP-12 microarray. Microarray results from 10 samples were excluded due to insufficient call rates (i.e., lower than 0.6), resulting in 189 samples that were used for further analysis. These samples were genotyped using the hg19 human genome build as a reference and variant allele frequencies were calculated for a total number of 261,079 SNPs for which genotyping information was available (Additional file 1: Table S1).

**Fig. 2:**
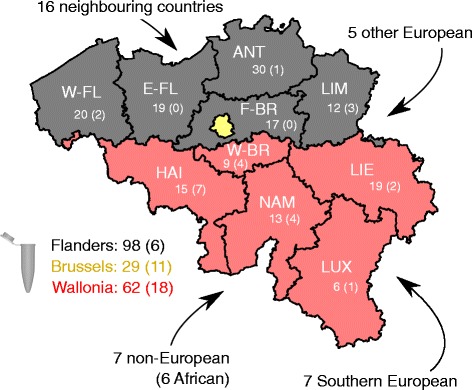
DNA sampling in the BelPHG-21 study, Belgium 2016. DNA was sampled and analyzed from 189 NHIS-2013 participants. The number of collected samples is indicated for the three regions and 10 provinces. Numbers between brackets indicate the number of samples collected from participants with a migration background. Main origins from migrants are indicated by the arrows. See Additional file 2: Table S2 for more details and province abbreviations

Belgium is composed of three different geographical regions: Flanders, Brussels, and Wallonia. The 189 samples that were used for analysis in this study were donated by volunteers in all three regions: 98 from Flanders, 62 from Wallonia, and 29 from Brussels (Fig. 2). These numbers are proportional to the population size of the respective regions with an overrepresentation from the Brussels region (15.3, 17.3, and 24.7 samples per million inhabitants for Flanders, Wallonia, and Brussels respectively) (Additional file 2: Table S2). This oversampling of the Brussels region is related to the way the NHIS was constructed [11]. Similarly, the distribution of these samples was relatively proportional to the population size from each of 10 provinces in Belgium with a variation from 11.3 to 26.7 samples per million inhabitants (Fig. 2 and Additional file 2: Table S2).

From the samples used for analysis, 35 (18.5%) were donated by an individual with non-Belgian roots (here defined as an individual whose mother and/or father was born outside Belgium, as registered in NHIS). These individuals mainly originated from neighboring countries (16), Southern Europe (7), and Africa (6). Proportionally, the highest numbers of non-Belgian samples were obtained from individuals living in Brussels (11/29 samples; 37.9%), followed by Wallonia (18/62; 29.0%), and Flanders (6/98; 6.1%) (Fig. 2). These numbers are representative for the current structure of Belgian population, with important migration waves in the past 50 years, mainly from Southern Europe and Northern Africa, and with the highest immigration numbers found in the Brussels region [13].

### The Belgian population is a typical European population with genetic signals of recent migration from the African continent

**Fig. 3:**
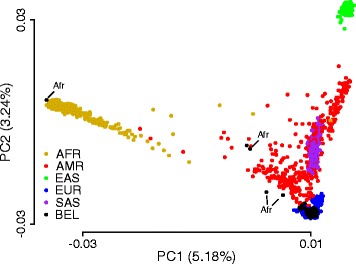
Genetic variation of the Belgian population related to continental populations. Plot of the first two principal components (PC) based on a PC analysis using 47,802 SNPs from five continental populations as indicated (AFR African, AMR American, EAS East Asian, EUR European, SAS South Asian). Belgian data (BEL) were mapped independently. Total variability captured by each PC is indicated by axis labels. For Belgian samples with a migration background that map outside the European population, the background is indicated. See main text for SNP filtering criteria

We first compared the genetic structure of the Belgian population with other populations worldwide. To restrict this analysis to the most informative and independent SNPs, only SNPs located on autosomes with a minor allele frequency of at least 5%, less than 2% missing values, and a linkage disequilibrium lower than 0.2 were used for structural comparison. These filtering criteria resulted in a selection of 47,802 SNPs. These SNPs were used for a principal component (PC) analysis using continental population data (i.e., African, American, South Asian, East Asian, and European populations) from the 1000 Genomes Project [1, 2, 14]. The main variance was captured by the first four PCs (10.4%, Additional file 3: Figure S1a). Using the first two PCs, which capture 5.2 and 3.2% of the total variance respectively, an expected and clear separation was observed between the different continental populations, with the largest differences between African, East Asian, and European populations (Fig. 3). By applying this PC model on the Belgian SNP genotype data, 184/189 samples were perfectly mapped on the European population. The remaining five samples were mapped towards or on the African population, and for four of these samples, an African origin was indeed confirmed in the NHIS data (Fig. 3 and Additional file 3: Figure S1b). These findings show that the genetic structure of the Belgian population is a typical European population with signals of recent migration from the African continent.

### The genetic structure of the Belgian population mirrors its geographical location in Europe

**Fig. 4:**
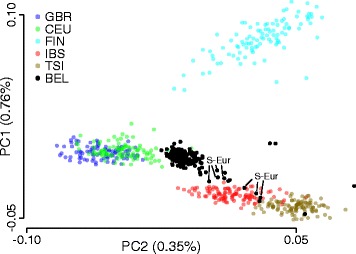
Genetic variation of the Belgian population related to other European populations. Plot of the first two principal components (PC) based on a PC analysis using 41,083 SNPs from five other European populations as indicated (GBR British, CEU Utah Residents with Northern and Western European Ancestry, FIN Finnish, IBS Iberian population in Spain, TSI Toscani in Italy). Belgian data (BEL) were mapped independently. Total variability captured by each PC is indicated by the axis labels. All Belgian samples with a South European migration background are indicated. See Additional file 4: Figure S2 for more details related to migration backgrounds. See main text for SNP filtering criteria

Our next goal was to examine how the Belgian population differs from other European populations. After a similar SNP filtering strategy as described above, the PC analysis was repeated using the European populations from the 1000 Genomes Project. Only the first two PCs were informative for a rather small variance of 0.76 and 0.35% respectively (Additional file 4: Figure S2a). As expected, the PC plot mimics European geography with a clear separation between Finnish, British, and South European populations. In agreement with this geographical orientation, most of the Belgian population mapped between the British and Southern European populations. Few samples mapped closer or within the South European populations, and for seven of these, a South European origin could indeed be confirmed (Fig. 4 and Additional file 4: Figure S2b). These data show the uniqueness of the Belgian genetic structure in Europe with geographically related differences with the other European populations.

### Minor genetic differences between Flanders and Wallonia

**Fig. 5:**
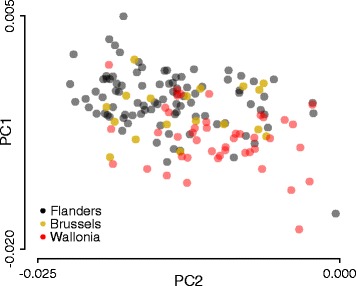
Regional genetic variation within the Belgian population. Belgian samples were mapped on the European PC model (41,083 SNPs) and colored based on the region of inhabitance as indicated

To examine potential differences between the three main geographic regions in Belgium, we excluded the 35 samples with non-Belgian origins and indicated regional information on the European PC model (Fig. 5). This PC analysis demonstrates a minor but clear genetic substructure in the Belgian population along a north-west to south-east geographic gradient, with Flemish inhabitants mapping in the north-west, Walloon inhabitants mapping in the south-east, and individuals from Brussels in between. A similar analysis based on sample origin information from the 10 different provinces confirmed these geographic-related genetic differences (Additional file 5: Figure S3).

To quantify the genetic differences between the three Belgian regions and compare them with other European populations, we calculated fixation indices (i.e., Fst values). Fst is a metric with values between 0 and 1 that is used to measure genetic distance between populations, in which high pairwise values indicate large population differentiation and vice versa. Fst values between the three Belgian regions were very small (between 1.8 and 2.5e−04) and smaller than with any other European population (Fst 3.2e−04 or higher) (Table 2), confirming the results from the PC analyses.

**Table 2:** Fst pairwise genetic distances between Belgian regional and other European populations, BelPHG-21 study, Belgium 2016

	GBR	FIN	IBS	CEU	TSI	Flanders	Wallonia	Brussels
GBR	0							
FIN	6.7e−03	0						
IBS	2.4e−03	1.0e−02	0					
CEU	3.2e−04	6.2e−03	2.2e−03	0				
TSI	3.7e−03	1.1e−02	1.5e−03	3.3e−03	0			
Flanders	6.5e−04	6.7e−03	1.9e−03	3.2e−04	2.7e−03	0		
Wallonia	7.1e−04	7.1e−03	1.4e−03	4.1e−04	2.1e−03	2.5e−04	0	
Brussels	6.5e−04	7.0e−03	1.8e−03	4.3e−04	2.5e−03	1.8e−04	1.8e−04	0

Finally, we correlated the variant allele frequencies of the Belgian population with other European populations. This analysis also allowed us to compare the Belgian population to the geographically and linguistically related population of the Netherlands, for which allele frequency data, but no genotyping data, are publicly available (Genome of the Netherlands project, GoNl [6]). As expected, and similar to the PC and Fst analysis, extremely good correlations were found with all European populations (Pearson correlation coefficients of 0.972 and higher). The best correlation was found with the GoNl data (Pearson correlation coefficient of 0.992; Additional file 6: Figure S4).

In summary, these data show minor but clear genetic differences between the different regions in Belgium with a north-west to south-east geographical correlation. However, these differences were smaller than with any other European population.

## Discussion

The BelPHG-21 study aims at developing an approach to estimate genetic variability at the level of the Belgian population and exploring how this genetic information can be associated with information collected via NHIS questionnaires. Here, we reported on the genetic structure of the Belgian population using ± 300,000 genome-wide SNPs, derived from saliva DNA sampled from 189 participants from the latest NHIS (2013). This is the first study on the genetic structure of the Belgian population and its regional differences. The Belgian population was found to be a typical European population with signals of recent migration from the African continent. Within Europe, the Belgian population has its unique properties, which clearly mirrors the geographic orientation and again with signs of recent migration from Southern Europe. While minor genetic differences were observed between the different Belgian regions, these were smaller than with any other population studied.

The linkage possibilities to health, demographic, and related data from the NHIS provide a unique opportunity to study the different factors that determine participation in a genetic study and to better understand public willingness to participate in population genetic research. Consent rates to participate in this study were rather low (14%). Related studies that recruited people with previous participation in health research reported on variable consent rates between 21 and 85% [15–18]. The 3-year time gap between the NHIS and the second contact, inviting participation in the BelPHG-21 study, may be a reason for the relatively low participation rate. Furthermore, subjects were contacted only by an invitation letter (and reminder). We noted regional differences in the consent rate, a higher consent for non-smokers and a positive association with age and education level. The higher likelihood of educated people to give consent is in agreement with other studies [19–21]. The correlation with age is more ambiguous, with reports of both younger age as well as older age being associated with lower consent [15, 20, 22]. The observation of smokers to be less likely to provide consent is in contradiction to other studies [23]. However, in our study, the difference was attributed to a higher consent rate for former smokers than for current smokers, while no significant difference was observed between current smokers and never smokers, indicating a more complex association with smoking status.

The close mirroring of population genetic variation with geography is a well-known phenomenon that has been described at the European level previously [8, 9]. Novembre et al. described a geographical map of Europe that arose naturally as an efficient PCA-based two-dimensional summary of genetic variation in European individuals from different countries (including 43 Belgians) [8]. This has been shown not only on national, but also on regional levels, as exemplified by genetic substructures that were observed in Sweden [10, 24], Finland [7, 25], the Netherlands [6], and several other European countries [9]. While this mirroring of the Belgian genetic structure with its geographical orientation in Europe was expected, it is quite remarkable to see the same phenomenon at regional and to a lesser extent even at provincial levels in a small country like Belgium.

Belgium is a country of immigration, with substantial labor-related migration waves after the Second World War [13]. This is reflected in this study, in which 18.5% of the samples originated from individuals with a migration background. Apart from the expected origin from neighboring countries, the most frequent migration backgrounds were South European and African, in agreement with migration data. This non-Belgian origin was clearly reflected in the genetic structure of the Belgian population. Information on migration background was derived from NHIS data and based on to the country of birth of the parents of the study participant. While this study demonstrated the usefulness of this type of NHIS-related information, there are some limitations. First, the information is limited to the first generation only (parents). This likely explains why one individual with a clear non-European genetic background was not found to have a migration background based on our criteria, which is unlikely. Secondly, there is also a risk of false positive migration findings as country of birth does not necessarily imply the individual (or his parents) originated in that country.

The NHIS contains information related to health, lifestyle, environment, etc. [11]. Therefore, linking genetic information to NHIS data has great potential for genome-wide association studies. While the current sample size (189) is rather limited, we will use the experience gathered in this study to set up a larger genetic study linked to the next NHIS (2018) where we aim to sequence the entire genome from a representative sample of ± 1000 individuals from the Belgian population using whole genome sequencing techniques. This genetic epidemiology study will also give a concrete idea about the expected number of harmful variants in the genome of a typical Belgian individual, including the prevalence of several genetic disease carriers. Results from these studies will be an invaluable resource in the transition towards precision public health, focusing on subsets of the population at increased risk, rather than on the entire population.

## Conclusions

This is the first study on the genetic structure of the Belgian population and its regional variation. The Belgian population is a typical European population with minor but clear differences between the regions and clear signs of recent migration.

## Methods

### BelPHG-21 study design

The BelPHG-21 study is a cross-sectional population-based study, conducted in 2016. The study population was selected from the participants of the most recent (2013) NHIS. Persons from the German community or with a missing national identification number were excluded for logistic reasons. Only one member per household was included, and the participants that did not complete the auto-questionnaire part of the NHIS in 2013 (which contains information on health, lifestyle, and environment), that were deceased, deregistered or with a discrepancy between self-reported and calculated age were excluded. From the resulting 5768 eligible candidates, a random subsample of 1468 individuals was invited to participate in the BelPHG-21 study. Individuals were invited via postal mail. They received an invitation letter with all the information on the BelPHG-21 study as well as informed consent forms to return in case of participation. Individuals that consented to participate in the study were subsequently sent a saliva sample collection kit (Oragene® saliva collection device (OG-500, DNA Genotek Inc. Ottawa, Canada)) accompanied by an information letter, a user manual for sample collection, a safety bag, and a pre-paid and UN3373-labeled bubble. Each package complied with the guidance on regulations for the transport of infectious substances. All communication with study participants (consent, sampling kits) was performed by a trusted third party (Belgian Federal Service of Internal Affairs) that was not involved in the downstream research in any way. They submitted the received samples to the BelPHG-21 research team and provided linking information to the NHIS database.

### Sample preparation and SNP array

Upon receipt of saliva samples by the BelPHG-21 research team, DNA was manually extracted from the total sample following the manufacturer’s instructions (DNA Genotek, PD-PR-015 Issue 10/2015-01). DNA samples were quantified using UV absorbance, and SNPs at ± 300,000 sites were determined using the whole genome scanning 12-sample Illumina HumanCytoSNP-12v2.1 BeadChip according to the manufacturer’s instructions. Images were captured on Cytoscan (Illumina), and data were primarily analyzed using Illumina’s GenomeStudio software.

### NHIS data

Information related to the study participants’ residence (region and province), migration background, and a set of variables that could influence the consent rate was obtained from the NHIS-2013 database. A subject was considered to have a migration background when at least one of its parents was born outside of Belgium. The variables that were considered to potentially influence consent rates were the background characteristics (demographic information, education, employment, income), health behavior and lifestyle characteristics (substance abuse, nutritional status, physical activity), physical and social environment characteristics (housing, passive smoking), and health and wellbeing characteristics (chronic diseases, mental health, perceived health). Selection of the variables for the predictive model was conducted by stepwise-weighted binomial regression analysis. Survey weights were calculated based on age, gender, and region of residence.

### Demographic and geographic data

Demographic data from the 2013 Belgian population were downloaded from Eurostat (http://ec.europa.eu/eurostat). Belgian geographic data were obtained using the BelgiumMaps.Statbel R package (available at http://www.datatailor.be/rcube/).

### SNP data processing

Genotype calls were imported in R and converted in a *sample x SNP* matrix. Samples with genotyping call rates lower than 0.6 were excluded from further analysis. All genotypes were converted so that the reference allele always referred to the positive strand of the human genome build hg19. Genomic coordinates for all SNPs were retrieved from dbSNP Build 144 [26]. SNPs not present in dbSNP were excluded from further analysis. The R SNPRelate package [27] was used for downstream analysis (calculation of allele frequencies, principal component analysis, …). Therefore, genotyping data were first converted in genomic data structure (gds) format.

### Principal component analysis

A principal component (PC) analysis was performed using phase 3 data from 1000 genomes [2]. VCF and sample information data were downloaded from the 1000 genomes ftp site (ftp://ftp.1000genomes.ebi.ac.uk/vol1/ftp/release/20130502/), and genotype information from all SNPs overlapping with the BelPHG-21 study was extracted using the R VariantAnnotation package [28]. Belgian data were mapped independently on the PC model. The PC analysis was performed using the most informative SNPs by applying the following filtering criteria: (1) SNPs measured in all datasets, (2) lower than 2% missing values in both the 1000 genome dataset that was used to perform the PC analysis and the Belgian data, (3) only autosomes, (4) minor allele frequency higher than 5%, and (5) linkage disequilibrium lower than 0.2.

### Variant allele frequency correlations

Correlations between variant allele frequencies (VAF) were determined using Pearson’s correlation. Correlations were determined for SNPs after filtering using the higher mentioned criteria. Variant allele frequencies from the Genome of the Netherlands (GoNl) project were derived from the release 5 vcf files downloaded at https://molgenis26.target.rug.nl/downloads/gonl_public/variants/release5/.

## Additional files

Additional file 1: Table S1. SNP variant allele frequencies of the Belgian population, BelPHG-21 study, Belgium 2016. Variant allele frequencies (VAF) for all SNPs were calculated with reference to the human genome build hg19 on all samples or after exclusion of samples with a foreign origin (indicated by tab names). Columns indicate SNP ids, chromosome, position, reference allele, variant allele, VAF, and frequencies of homozygous reference (AA), heterozygous (AB), and homozygous variant (BB) alleles respectively. (XLSX 28534 kb)

Additional file 2: Table S2. Population details of the samples analyzed in the BelPHG-21 study, Belgium 2016. Indication of the number of samples taken from NHIS-2013 participants in each region and province, total number of inhabitants in 2013, number of samples taken per million inhabitants, number of invited participants, and sampling percentages. (XLSX 10 kb)

Additional file 3: Figure S1. Genetic variation of the Belgian population related to continental populations. Principal component (PC) analysis using 47,802 SNPs from five continental populations with mapping of the Belgian population. (a) Screeplot showing the variability captured by the first 10 PCs. (b) Pairwise plots of the first four PCs. Populations are indicated below the plot (AFR: African; AMR: American; EAS: East Asian; EUR: European; SAS: South Asian; BEL: Belgian). (PDF 7654 kb)

Additional file 4: Figure S2. Genetic variation of the Belgian population related to other European populations. Principal component (PC) analysis based on 41,083 SNPs from five different European populations (see Fig. 4 for details). (a) Screeplot showing the variability captured by the first 10 PCs. (b) PC plot of five European populations with mapping of the Belgian population and indication of their migration backgrounds. For visualization purposes, the other European populations are shown in gray. (PDF 366 kb)

Additional file 5: Figure S3. Provincial genetic variation within the Belgian population. Belgian data were mapped on the European PC model (41,083 SNPs). Panels show plots of the first two PCs with indication of the province of inhabitance as indicated. The central map of Belgium shows the geographical location of each province. Panels with Flemish provinces are shown on top while Walloon province panels are shown on the bottom. (PDF 1806 kb)

Additional file 6: Figure S4. Variant allele frequency correlations between Belgian and other populations. Variant allele frequencies (VAF) for all SNPs were calculated with reference to the human genome build hg19. Plots show the VAF of Belgian versus other continental (a) and European (b) populations. Pearson correlation coefficients are shown on top of each plot. For visualization purposes, only 1000 random points are shown. (PDF 5259 kb)

## Abbreviations

PC: Principal component
SNP: Single nucleotide polymorphism
VAF: Variant allele frequency
