# Supplementary figures and images for: The genetic structure of the Belgian population
Source: Hum Genomics. 2018 Feb 2;12:6. doi: 10.1186/s40246-018-0136-8 (PMC5796395; doi:10.1186/s40246-018-0136-8)

**A**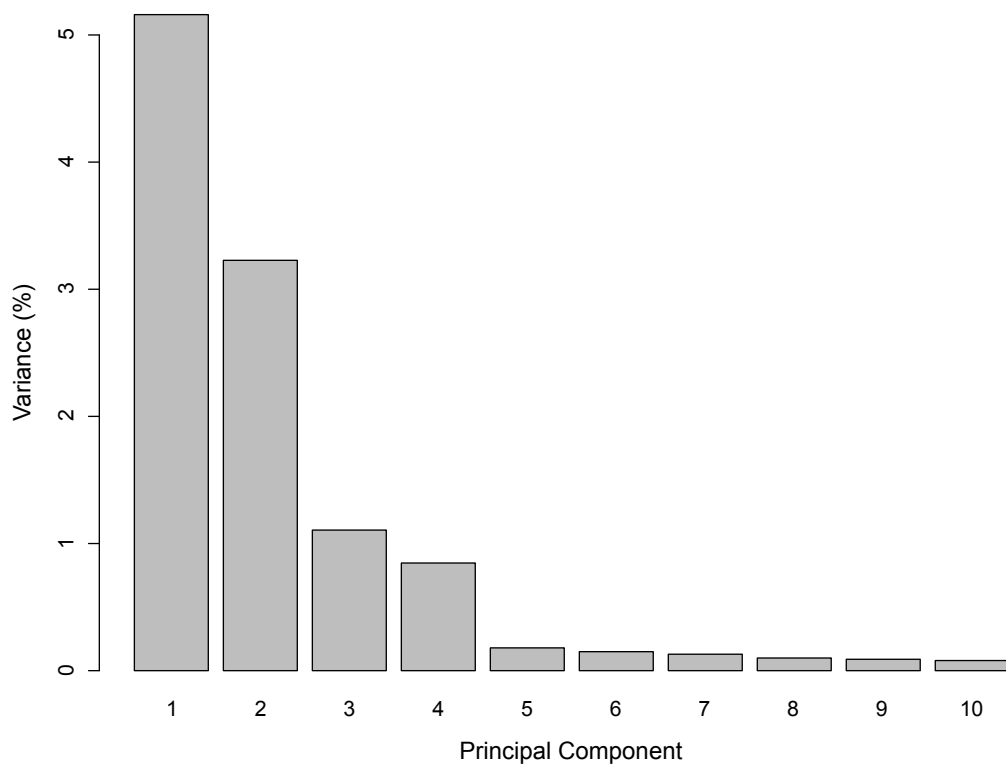**B**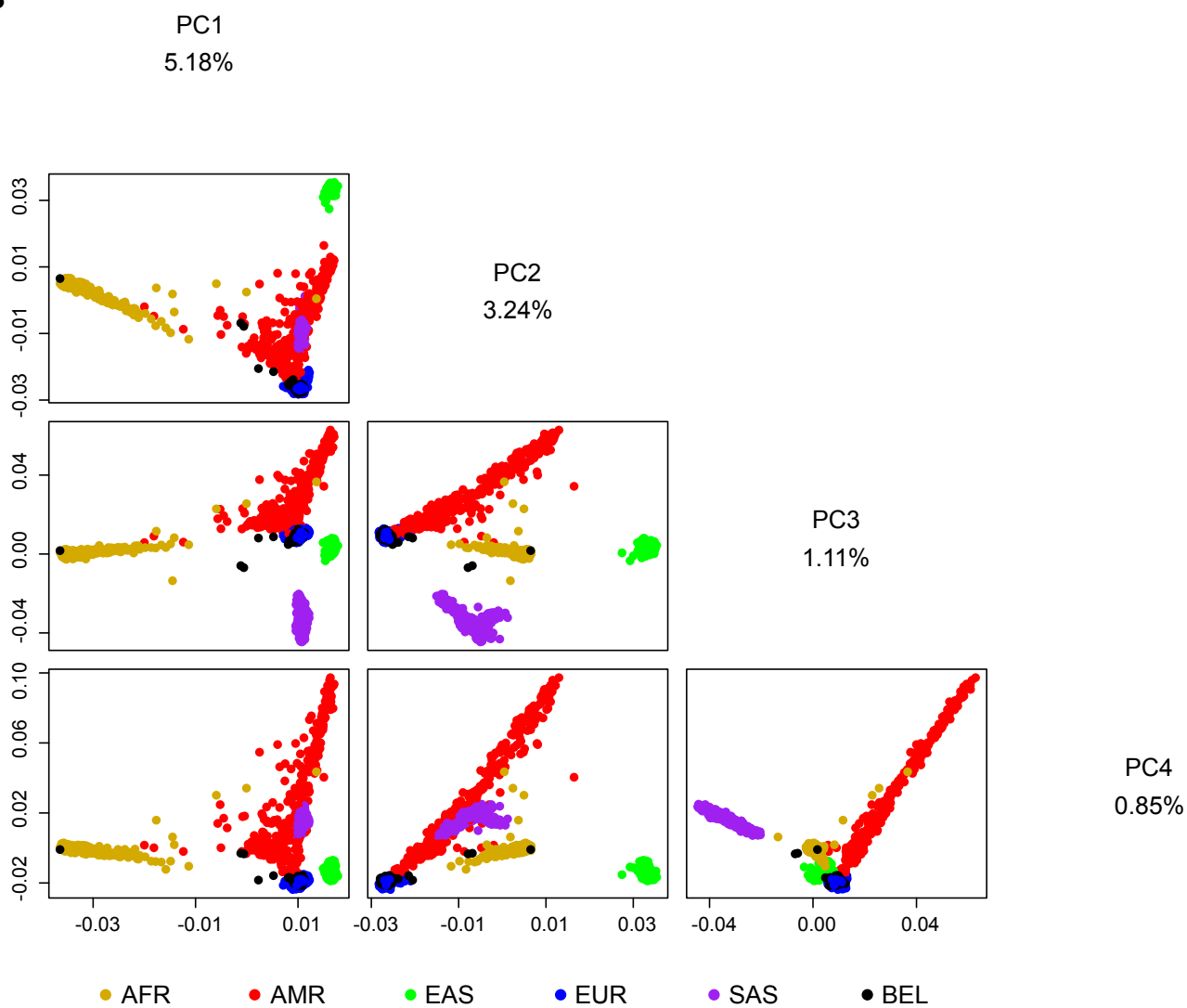

Supplement: Supplementary file 3 — Genetic variation of the Belgian population related to continental populations. Principal component (PC) analysis using 47,802 SNPs from five continental populations with mapping of the Belgian population. (a) Screeplot showing the variability captured by the first 10 PCs. (b) Pairwise plots of the first four PCs. Populations are indicated below the plot (AFR: African; AMR: American; EAS: East Asian; EUR: European; SAS: South Asian; BEL: Belgian). (PDF 7654 kb) [file 40246_2018_136_MOESM3_ESM.pdf]

**A**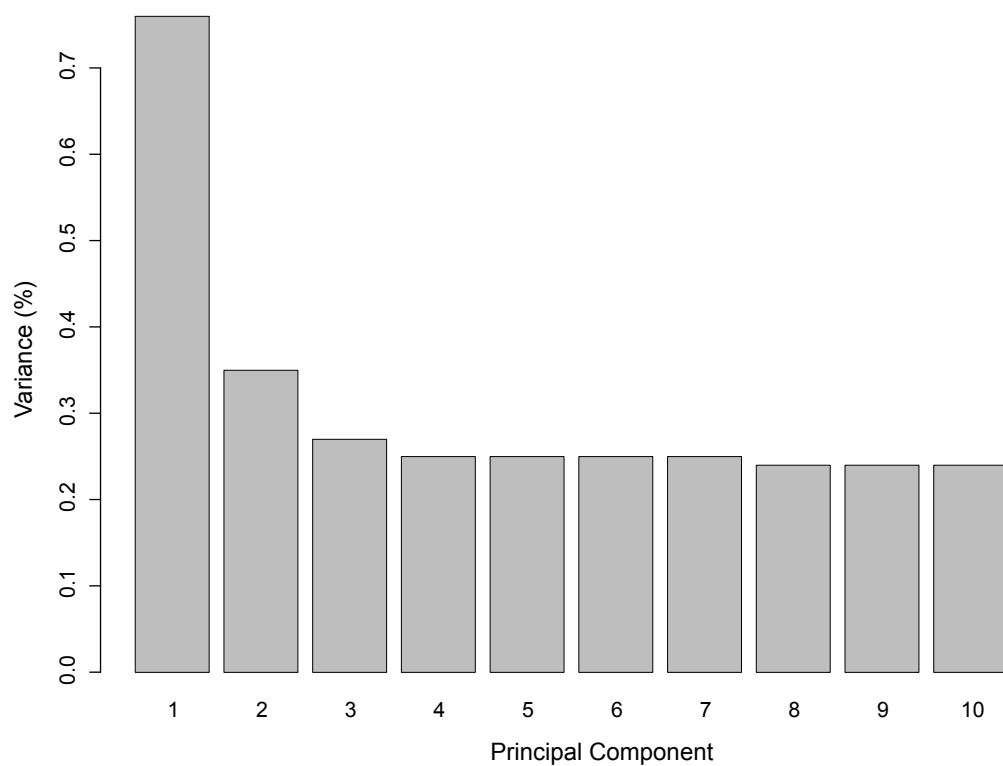**B**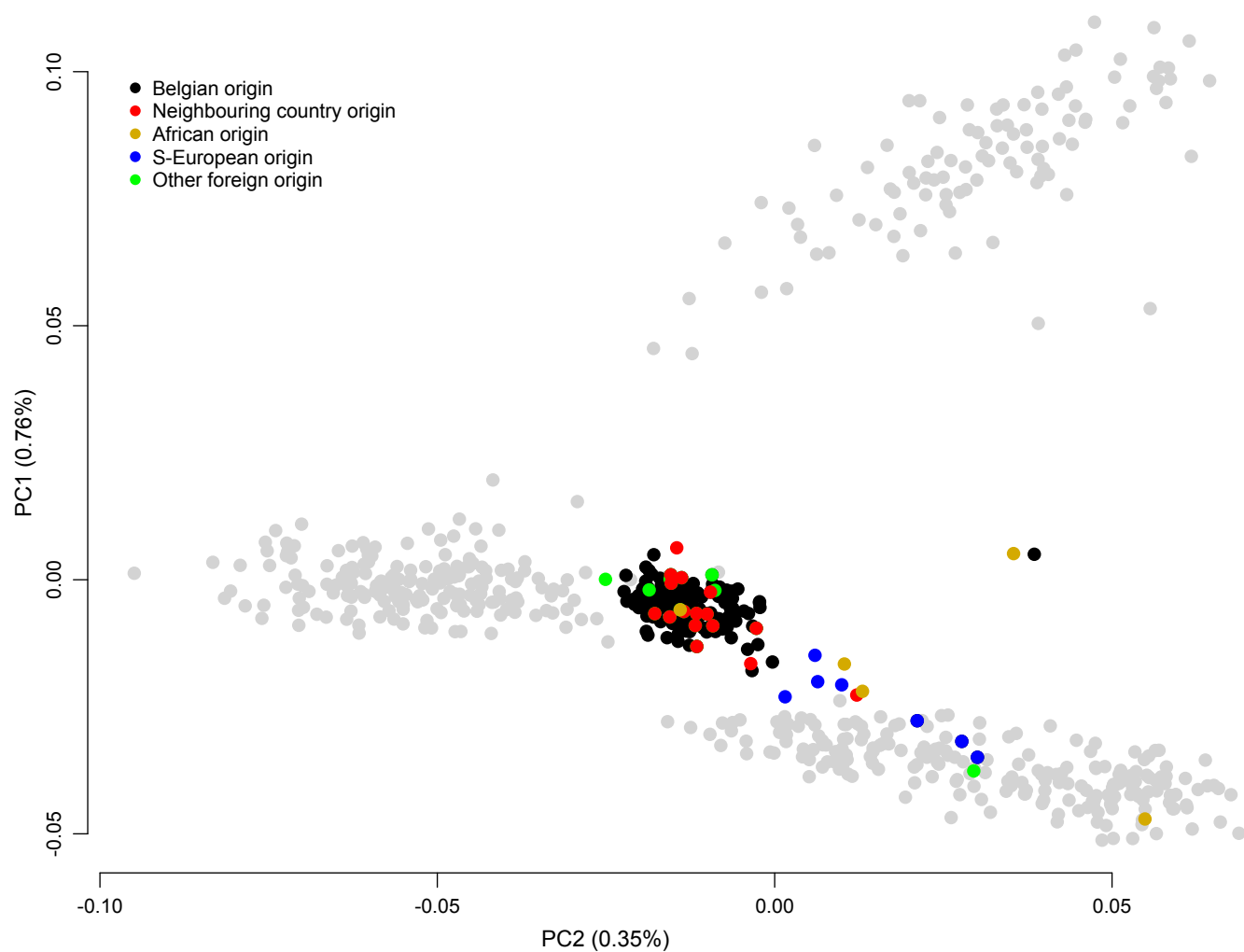

Supplement: Supplementary file 4 — Genetic variation of the Belgian population related to other European populations. Principal component (PC) analysis based on 41,083 SNPs from five different European populations (see Fig. 4 for details). (a) Screeplot showing the variability captured by the first 10 PCs. (b) PC plot of five European populations with mapping of the Belgian population and indication of their migration backgrounds. For visualization purposes, the other European populations are shown in gray. (PDF 366 kb) [file 40246_2018_136_MOESM4_ESM.pdf]

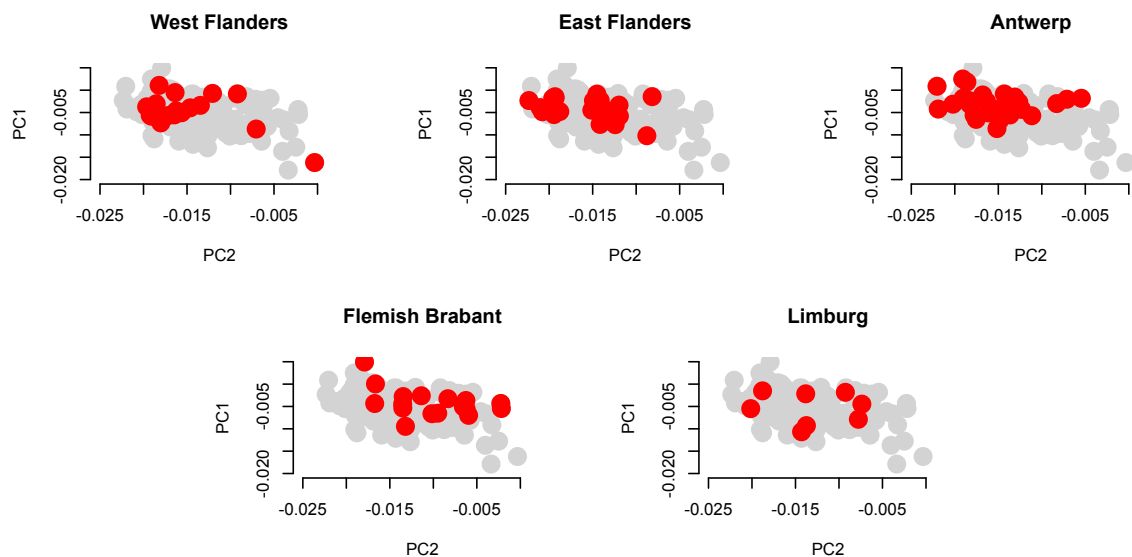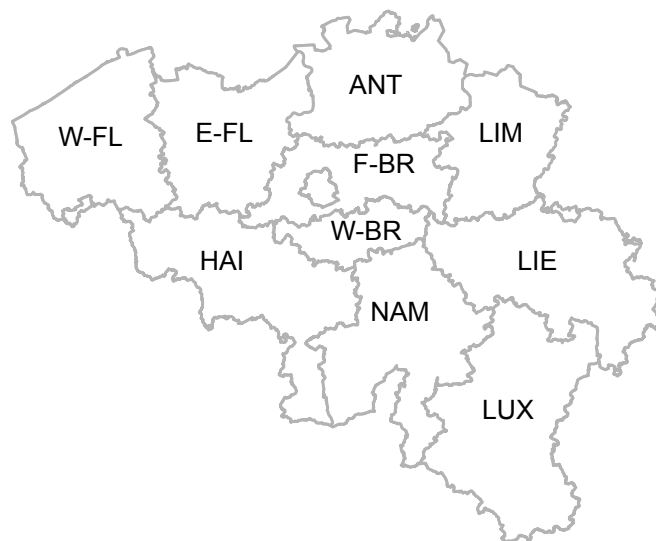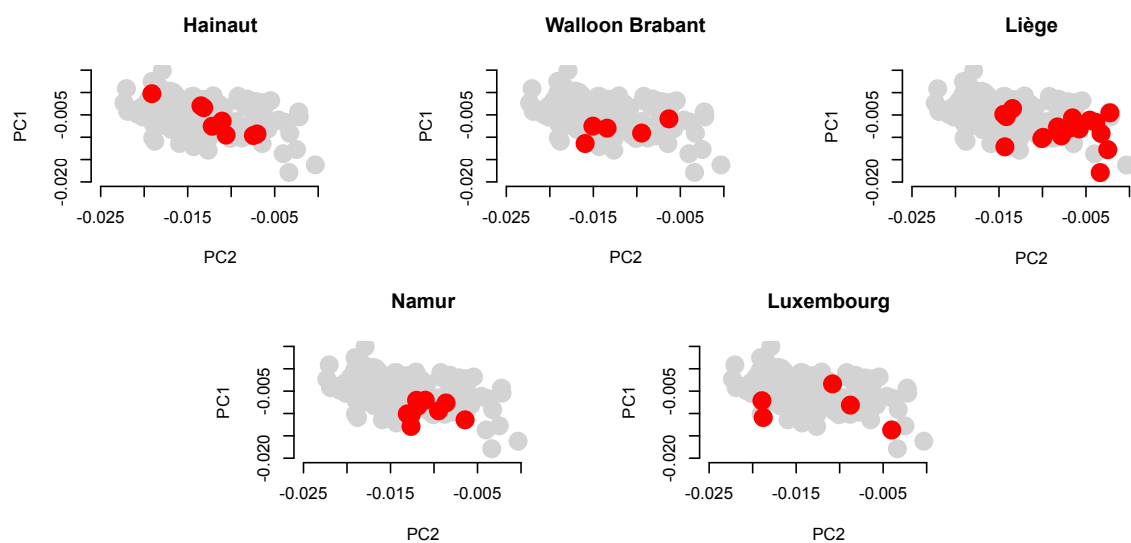

Supplement: Supplementary file 5 — Provincial genetic variation within the Belgian population. Belgian data were mapped on the European PC model (41,083 SNPs). Panels show plots of the first two PCs with indication of the province of inhabitance as indicated. The central map of Belgium shows the geographical location of each province. Panels with Flemish provinces are shown on top while Walloon province panels are shown on the bottom. (PDF 1806 kb) [file 40246_2018_136_MOESM5_ESM.pdf]

**A**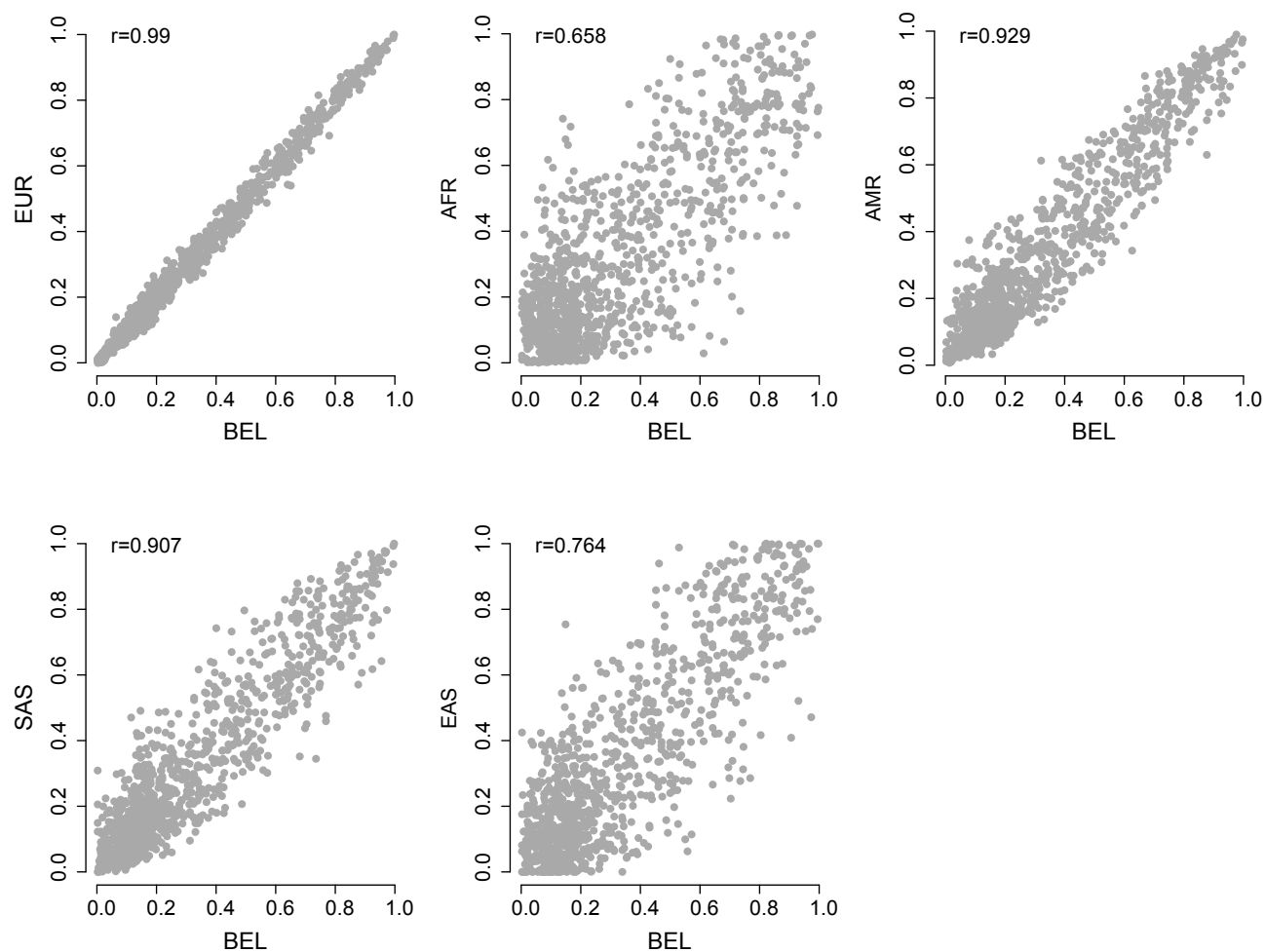**B**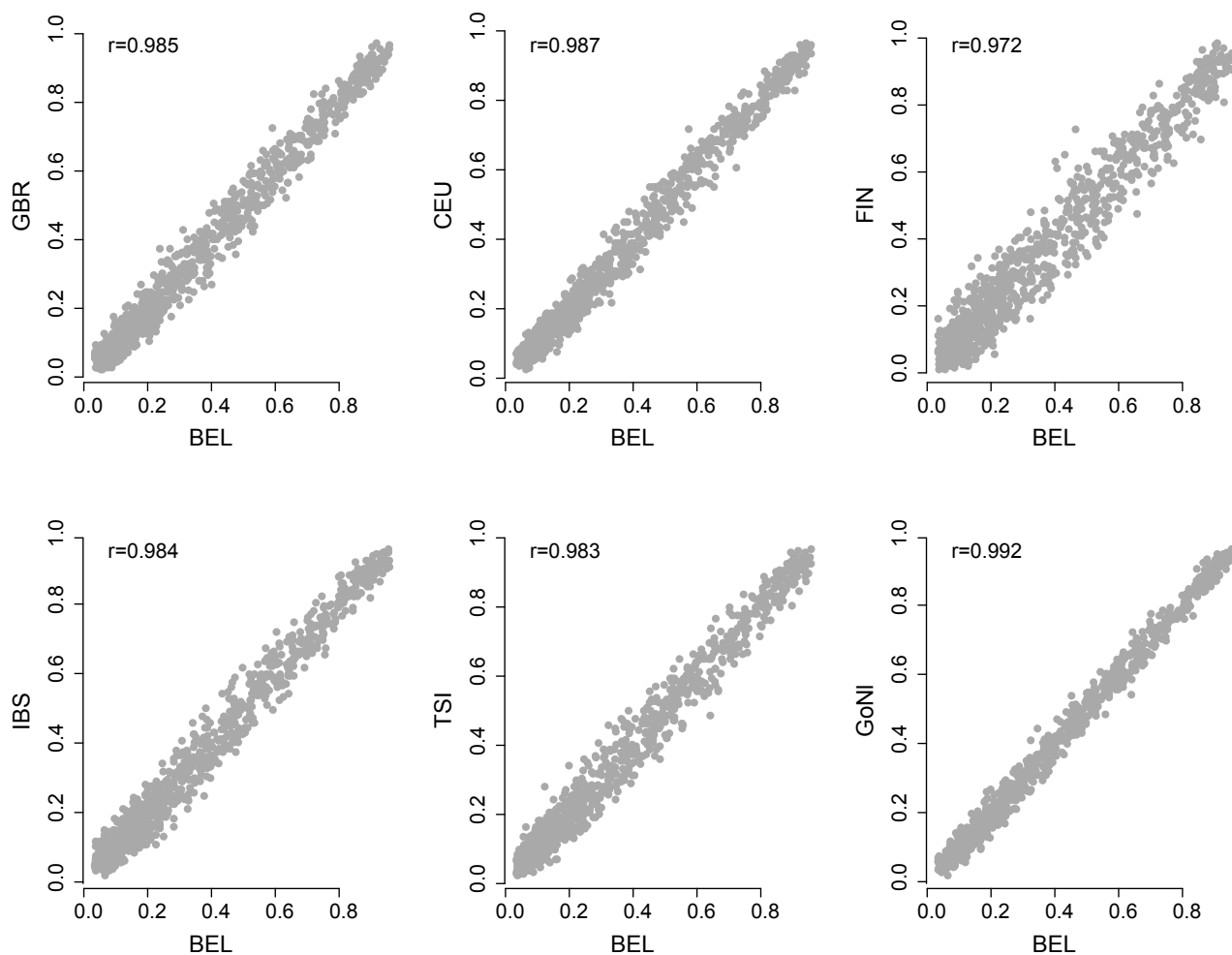

Supplement: Supplementary file 6 — Variant allele frequency correlations between Belgian and other populations. Variant allele frequencies (VAF) for all SNPs were calculated with reference to the human genome build hg19. Plots show the VAF of Belgian versus other continental (a) and European (b) populations. Pearson correlation coefficients are shown on top of each plot. For visualization purposes, only 1000 random points are shown. (PDF 5259 kb) [file 40246_2018_136_MOESM6_ESM.pdf]
